# Retraction: Aclarubicin regulates glioma cell growth and DNA damage through the SIRT1/PI3K/AKT signaling pathway

**DOI:** 10.1039/d1ra90038b

**Published:** 2021-01-26

**Authors:** Laura Fisher

**Affiliations:** Royal Society of Chemistry Thomas Graham House, Science Park, Milton Road Cambridge CB4 0WF UK advances-rsc@rsc.org

## Abstract

Retraction of ‘Aclarubicin regulates glioma cell growth and DNA damage through the SIRT1/PI3K/AKT signaling pathway’ by Jun-Feng Huo *et al.*, *RSC Adv.*, 2019, **9**, 28775–28782, DOI: 10.1039/C9RA05572J.

The Royal Society of Chemistry hereby wholly retracts this *RSC Advances* article due to concerns with the reliability of the data. The images in the article, and the raw data provided by the authors, were screened by an image integrity expert.

The published western blots in the pATM panel in Fig. 5E showed signs of manipulation. In addition, the raw data provided for all the western blots in the article was not genuine as it consisted of the bands being placed onto false backgrounds. Therefore, the raw data provided by the authors cannot be used to validate the published data.

Given the significance of the concerns about the validity of both the data in the article and the raw data provided by the authors, the findings presented in this paper are not reliable.

The authors have been informed but have not responded to any correspondence regarding the retraction.

Signed: Laura Fisher, Executive Editor, *RSC Advances*.

Date: 15^th^ January 2021.

## Supplementary Material

